# Supplementary material for: Tead1a Initiates Transcriptional Priming Through the TEAD1a/YAP‐Notch1‐Spi1/Cebpα Axis to Promote Neutrophil Fate
Source: Adv Sci (Weinh). 2025 Aug 28;12(41):e05441. doi: 10.1002/advs.202505441 (PMC12591127; doi:10.1002/advs.202505441)
Supplement: Supplementary file 4 — Supplementary Dataset [file ADVS-12-e05441-s004.zip › 3-5.zip!/3-5/02-5.cluster/cluster13/cluster13_featureplot.pdf]

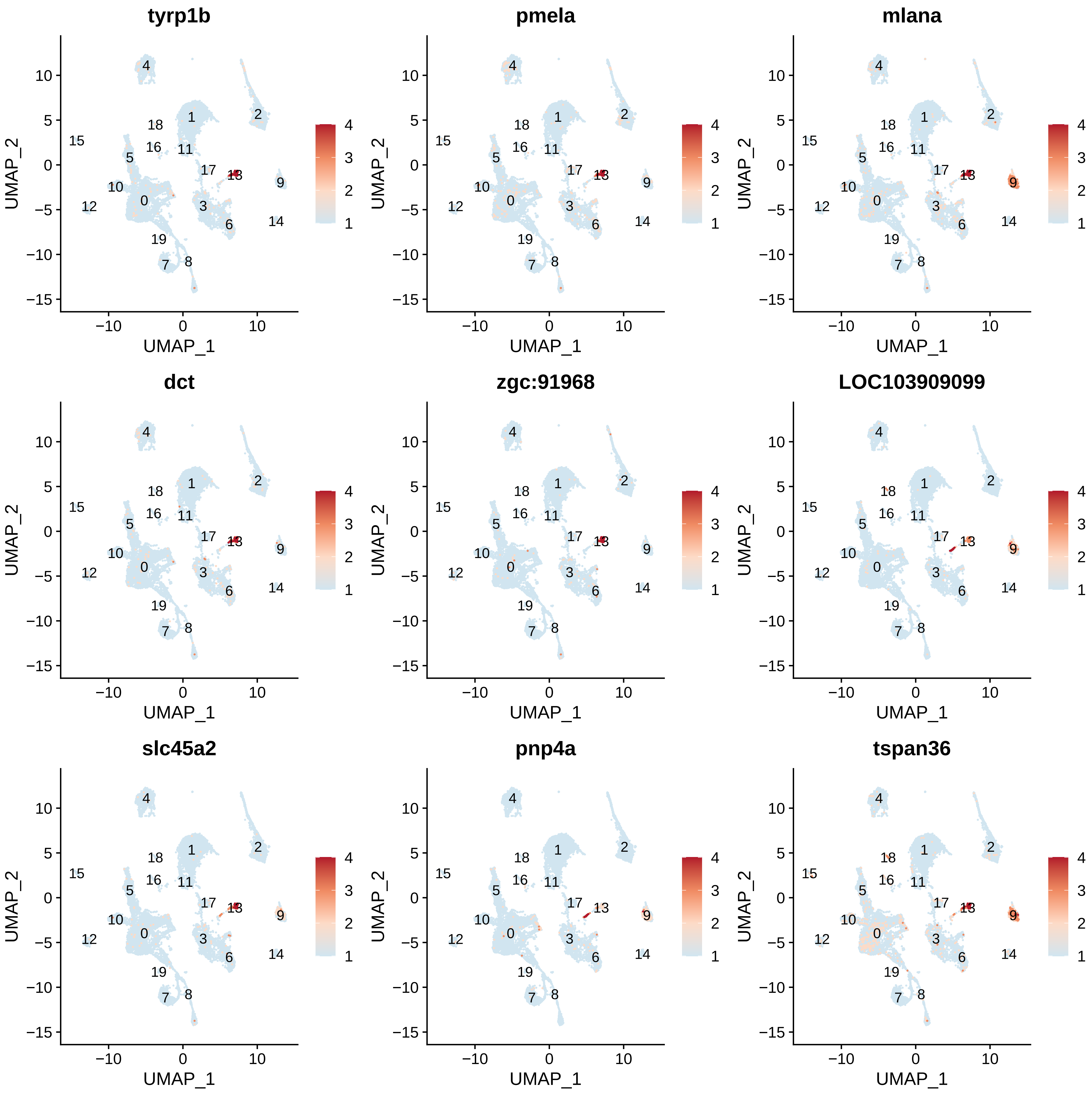
tyrp1b
pmela
mlana
dct
zgc:91968
LOC103909099
slc45a2
pnp4a
tspan36
UMAP_1
UMAP_2
0
1
2
3
4
5
6
7
8
9
10
11
12
13
14
15
16
17
18
19
